# Clinicopathological factors in bladder cancer for cancer-specific survival outcomes following radical cystectomy: a systematic review and meta-analysis

**DOI:** 10.1186/s12885-019-5924-6

**Published:** 2019-07-19

**Authors:** Lijin Zhang, Bin Wu, Zhenlei Zha, Wei Qu, Hu Zhao, Jun Yuan

**Affiliations:** 1 Department of Urology, Affiliated Jiang-yin Hospital of the Southeast University Medical College, Jiang-yin, 214400 China; 2 Department of Pharmacy, Affiliated Jiang-yin Hospital of the Southeast University Medical College, Jiang-yin, 214400 China

**Keywords:** Bladder cancer, Radical cystectomy, Cancer-specific survival, Meta-analysis

## Abstract

**Background:**

Assessing the prognostic significance of specific clinicopathological features plays an important role in surgical management after radical cystectomy. This study investigated the association between ten clinicopathological characteristics and cancer-specific survival (CSS) in patients with bladder cancer.

**Methods:**

In accordance with the Preferred Reporting Items for Systematic Reviews and Meta-analyses (PRISMA) guidelines, a literature search was conducted through the PubMed, EMBASE and Web of Science databases using appropriate search terms from the dates of inception until November 2018. Pooled hazard ratios (HRs) with 95% confidence intervals (CIs) were calculated to evaluate the CSS. Fixed- or random-effects models were constructed according to existence of heterogeneity.

**Results:**

Thirty-three articles met the eligibility criteria for this systematic review, which included 19,702 patients. The overall results revealed that CSS was associated with advanced age (old vs. young: pooled HR = 1.01; 95% CI:1.00–1.01; *P* < 0.001), higher tumor grade (3 vs. 1/2: pooled HR = 1.29; 95% CI:1.15–1.45; *P* < 0.001), higher pathological stage (3/4 vs. 1/2: pooled HR = 1.60; 95% CI:1.37–1.86; *P* < 0.001), lymph node metastasis (positive vs. negative: pooled HR = 1.51; 95% CI:1.37–1.67; P < 0.001), lymphovascular invasion (positive vs. negative: pooled HR = 1.36; 95% CI:1.28–1.45; P < 0.001), and soft tissue surgical margin (positive vs. negative: pooled HR = 1.42; 95% CI:1.30–1.56; P < 0.001). However, gender (male vs. female: pooled HR = 0.98; 95% CI: 0.96–1.01; *P* = 0.278), carcinoma in situ (positive vs. negative: pooled HR = 0.98; 95% CI: 0.88–1.10; *P* = 0.753), histology (transitional cell cancer vs variant: pooled HR = 0.90; 95% CI: 0.79–1.02; *P* = 0.089) and adjuvant chemotherapy (yes vs. no: pooled HR = 1.16; 95% CI: 1.00–1.34; *P* = 0.054) did not affect CSS after radical resection of bladder cancer.

**Conclusions:**

Our results revealed that several clinicopathological characteristics can predict CSS risk after radical cystectomy. Prospective studies are needed to further confirm the predictive value of these variables for the prognosis of bladder cancer patients after radical cystectomy.

**Electronic supplementary material:**

The online version of this article (10.1186/s12885-019-5924-6) contains supplementary material, which is available to authorized users.

## Background

Bladder cancer (BCa) is the most common malignancy of the urinary tract and occurs with a relatively high incidence in developing countries [1], with annual mortality rates ranging from approximately 1–5 deaths per 100,000 men and 0.5–1.5 deaths per 100,000 women [2]. Radical cystectomy (RC) with bilateral pelvic lymph node dissection is the gold standard for patients with localized muscle-invasive tumors. Despite a better understanding of BCa biology and the use of adjuvant therapies, BCa continues to have high mortality rates, and the oncological outcomes following RC have not changed in the last 30 years [3].

BCa prognoses vary widely. Many factors have been investigated as potential predictors of clinical outcome in BCa. Positive soft tissue surgical margins (STSM) [4], lymphovascular invasion (LVI) [5], lymph node metastasis (LNM) [6], concomitant carcinoma in situ (CIS) [7], and failure to receive adjuvant chemotherapy (ACT) [8] have been reported to be associated with poor prognoses for BCa after RC. Although these predictive variables have contributed to estimating the BCa recurrence risk and survival outcomes, additional variables that can integrate with well-established prognostic models and provide accurate risk grading for BCa patients after RC are critical.

A major problem for urologists is identifying prognostic factors that can predict cancer progression. The ability to determine cancer-specific survival (CSS) and provide integrated patient survivorship and better estimates of survival probability at each follow-up may lead to more informative prognostic information in patient monitoring [9].Therefore, we aimed to provide a comprehensive systematic review and meta-analysis of previous studies to investigate the prognostic roles of pathological status and clinical variables for CSS in patients following RC. We identified ten common clinicopathological characteristics that should be systematically assessed to guide postoperative decision-making after RC.

## Methods

### Search strategy

In line with the guidelines of Preferred Reporting Items for Systematic Reviews and Meta-analyses (PRISMA) [10], the electronic database of PubMed, EMBASE and Web of Science were searched for studies published prior to November 2018. The following search term combinations were used: ‘urinary bladder neoplasms’, ‘bladder and neoplasms’, ‘radical cystectomy’, ‘cancer-specific survival’, ‘clinical’, and ‘pathological’. The publication language was restricted to English. In addition, the reference lists of the identified studies were also searched manually.

### Inclusion and exclusion criteria

The inclusion criteria were as follows: (1) all patients with BCa were pathologically confirmed; (2) the study included prognostic factors for CSS following radical cystectomy; (3) treatment was limited to RC in all studies; and (4) the authors provided the hazard ratios (HRs) and 95% confidence intervals(CIs). The exclusion criteria were: (1) duplicates; (2) lack of sufficient data (HRs and CIs) for further analysis; and (3) case reports, reviews, letters, author replies, expert opinions or meeting abstracts. If the data overlapped across several different articles, only the most recent and informative article was selected.

### Data extraction and qualitative assessment

Two authors extracted the information from the selected studies. Any disagreement between the reviewers was resolved by discussion with a third author. The following information were collected from eligible studies: first author’s name, publication date, country, recruitment period, follow-up time, sample size, patient’s age, pathological stage, tumor grade, histopathological subtype in transitional cell cancer (TCC) and the HR and 95% CIs for CSS.

We evaluated the study quality using the 9-star Newcastle-Ottawa Scale (NOS) [11]. Scores of 7–9 indicated a high-quality study, and scores < 7 indicated a low-quality. The cohort study quality was assessed as follows: object selection, inter-group comparability, and outcome measurement. Dichotomous variables were presented as HRs with 95% CIs. If the data results were calculated by multivariate and univariate analysis simultaneously, the multivariate analyses were used.

### Statistical analysis

All calculations were performed using STATA 12.0 software (Stata Corp LP, College Station, TX, USA). Heterogeneity was estimated using the Higgins *I*-squared statistic test, and *P*_*heterogeneity*_ ≤ 0.1 or *I*^*2*^ > 50%. indicated heterogeneity among studies. When significant heterogeneity was observed among the studies, a random-effect (RE) model was used; otherwise, we adopted a fixed-effect (FE) model. To explore the source of heterogeneity, subgroup analysis was performed for CSS. Sensitivity analysis was conducted by excluding single studies one by one to examine the stability and reliability of the pooled results. A funnel plot and Egger’s test were used to statistically evaluate the publication bias between studies. Two-tailed *P* < 0.05 was considered statistically significant.

## Results

### Literature search

**Fig. 1:**
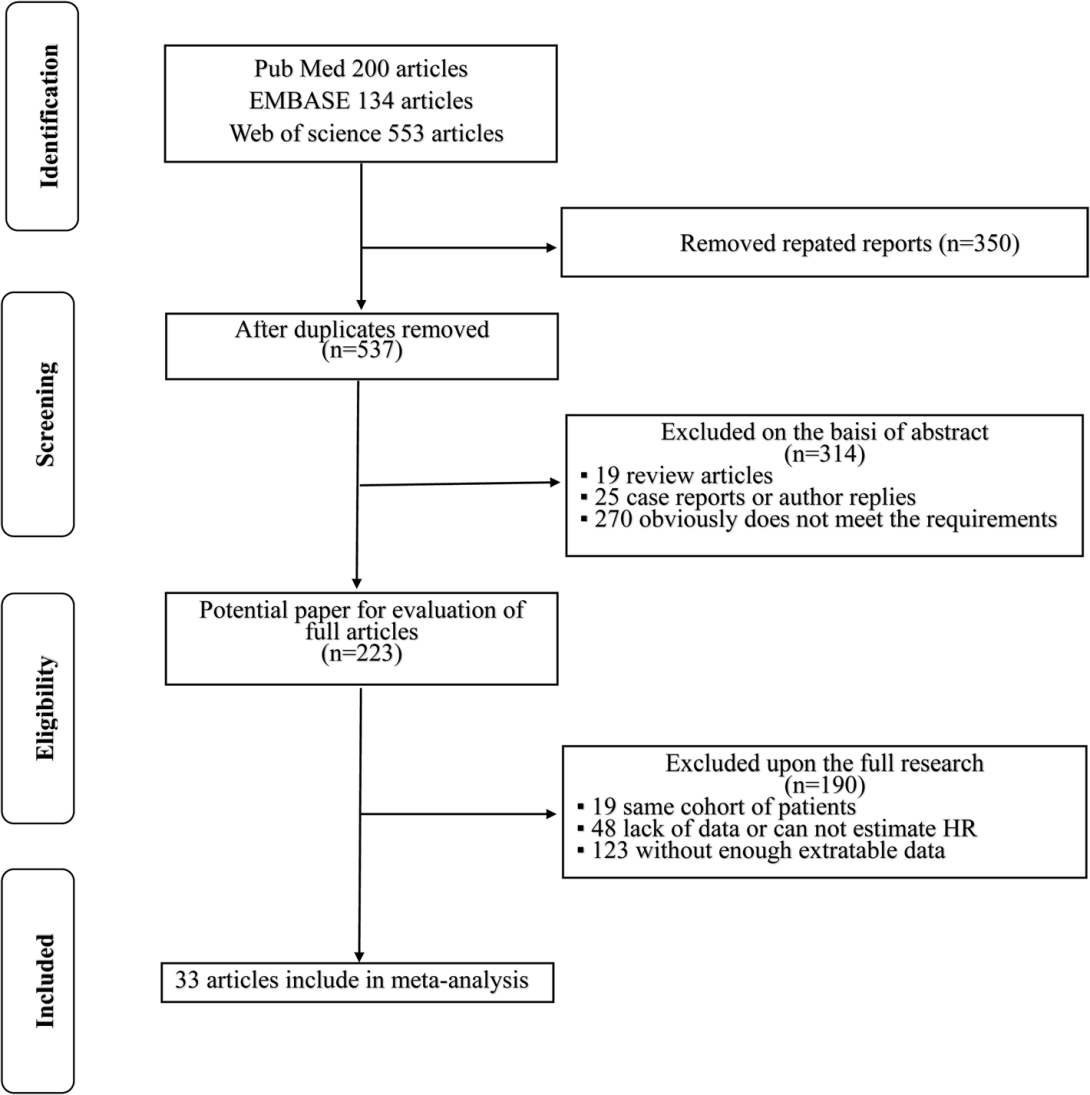
Flowchart of the literature search used in this meta-analysis

From the search criteria, 887 articles were identified from the databases and the manual search. Of these articles, 664 studies were excluded based on their titles and/or abstracts, resulting in 223 studies for further analysis. The full texts were then screened, and 190 papers were excluded because of insufficient survival information or duplicated cohorts. Finally, 33 studies [3, 5, 6, 8, 12–40] containing 19,702 patients (range 51–2,944) were included as per the eligibility criteria. Figure 1 presents a flowchart of the study selection process.

### Characteristics of eligible studies

Tables 1 and **2** summarize the main characteristics and clinicopathological outcomes of the 33 included studies. All studies were performed retrospectively, and all were published between 2007 and 2018. Of the included studies, 11 were conducted in Asia, 8 in Europe, 7 in North America, 4 at international multicenters, 3 in Turkey and 1 in Australia. Histopathological examinations were performed on resected tumor specimens. All studies used CSS as a common endpoint to evaluate the prognostic value of the clinicopathological indicators of survival. The quality scores of the studies ranged from 7 to 9.Therefore, all included studies were of high quality (studies with a score ≥ 7; Additional file 2: Table S1).

**Table 1:** Main characteristics of the studies included in the meta-analysis

Author	Year	Country	Recruitment period	No. of patients	Age (years)	Gender (m/f)	Follow-up (months)	Survival analysis
Mayr et al. [12]	2018	Muti-centers	2004–2014	500	Median (IQR) 72(65–78)	401/99	Median (IQR) 35 (20–58)	Age, gender, LNM, LVI, STSM, CIS, ACT
Hodgson et al. [13]	2018	Japan	1999–2005	235	Mean (range) 70.1 (46–93)	167/68	Median (range) 16 (1–206)	LNM, LVI, STSM
Muppa et al. [14]	2017	USA	1980–2010	965	Mean ± SD 67 ± 10.1	761/204	Mean 127.2	gender, LNM, LVI, STSM, histology, ACT
Li et al. [15]	2017	China	2004–2015	1,676	Mean (range) 66.4 (24–92)	1,376/300	Median (range) 78 (4–138)	Age, gender, grade, LVI
Kang et al. [16]	2017	Korea	1999–2012	385	Mean (range) 66 (59–72)	333/52	NA	Age, grade, stage, LNM, LVI, STSM,CIS
Gorgel et al. [17]	2017	Turkey	2006–2016	149	Mean ± SD 61.6 ± 9.13	139/10	NA	Age, gender, grade, stage, LNM, LVI
Andera et al. [18]	2017	USA	1988–2003	448	Median (IQR) 65(60–71)	373/75	Median (IQR) 170.4(122.4–205.2)	Age, gender, LNM, LVI, histology, ACT
Crozier et al. [19]	2017	Australia	2005–2014	220	Mean (range) 69.5 (60.3–74.9)	177/43	NA	gender, STSM
Morizawa et al. [20]	2016	Japan	2002–2013	110	Median (IQR) 72(65–76)	86/24	Median (IQR) 37.5 (11–65)	LNM, stage,
Liu et al. [21]	2016	China	2000–2013	296	Mean ± SD 61.7 ± 11.1	250/46	Median (IQR) 72.0 (49.0–121.0)	Age, gender, grade, LNM, ACT
Bostrom et al. [22]	2016	Muti-centers	1986–2008	581	NA	457/124	Median 68.4	Age, gender, grade, stage, LNM, ACT
Alimi et al. [23]	2016	France	1992–2012	331	Mean ± SD 65.7 ± 11.4	272/59	Median (range) 52.6 (6–267)	Age, LNM, LVI, ACT
Soave et al. [24]	2015	Germany	1996–2011	517	Median (IQR) 67(59–73)	400/117	Median (IQR) 45(21–83)	Age, gender, grade, LNM, LVI, STSM, CIS, ACT
Raza et al. [25]	2015	Muti-centers	2003–2015	702	Median (IQR) 69(61–76)	569/133	Median (IQR) 67(8–84)	Age, gender, stage, LNM, STSM, histology, ACT
Ozcan et al. [26]	2015	Turkey	1990–2013	286	Mean ± SD 60.7 ± 19.42	256/30	Median (range) 8 (0–144)	Age, gender, grade, stage, LNM, LVI, STSM, histology, CIS
Kwon et al. [27]	2015	Korea	1990–2012	746	Mean ± SD 62.4 ± 9.7	664/82	Median (range) 64.3 (1–231.4)	grade, stage, LNM, LVI, STSM, CIS
Kanatani et al. [8]	2015	Japan	1990–2012	61	Median (IQR) 64(59–75)	55/6	Median (IQR) 29(17–59)	Age, gender, grade, stage, LNM, LVI, STSM, ACT
Ferro et al. [28]	2015	Italy	2008–2013	1,037	Median (range) 70 (42–88)	804/233	Median (range) 22 (3–60)	Age, grade, CIS,ACT
Booth et al. [29]	2015	Canada	1994–2008	2,944	Median 69	2,107/695	NA	gender, stage, LNM, LVI, STSM, ACT
Albisinni et al. [30]	2015	Belgium	2000–2013	503	Median (IQR) 68 (62–74)	414/89	Median (IQR) 50(19–90)	LNM, STSM
Kawai et al. [31]	2014	Japan	1990–2005	84	Median (range) 65 (39–81)	70/14	NA	LNM, LVI
Kaushik et al. [32]	2014	USA	1980–2005	128	Median (IQR) 72 (64–74)	91/37	Median (IQR) 126(116.4–145.2)	gender, LNM, STSM, ACT
Brunocilla et al. [33]	2013	Italy	1995–2011	282	Median (IQR) 70 (63–75)	234/48	Mean (range) 59.2 (1–171)	Age, gender, grade, LNM, LVI, histology, ACT
Aziz et al. [3]	2013	Germany	2004–2010	150	Median (IQR) 70 (64–76)	121/29	Median (IQR) 46 (31–62)	Age, gender, grade, stage, LNM, LVI, CIS,ACT
Otto et al. [34]	2012	Germany	1989–2008	2,483	Median (IQR) 66.4(60.1–72.5)	1,976/507	Median (IQR) 42(21–79)	Age, grade, stage, LNM, LVI, CIS, ACT
Gondo et al. [35]	2012	Japan	2008–2009	194	Mean (range) 68(38–85)	162/32	Mean (range) 26.8 (3.1–131.8)	gender, stage, LVI, STSM
Yafi et al. [36]	2011	Muti-centers	1998–2008	2,287	Median (range) 68(26–90)	1,803/484	Median (IQR) 29.3(9–50)	Age, gender, grade, stage, LNM, STSM, histology, ACT
Faba et al. [37]	2011	Spain	1978–2002	141	Median (range) 63 (47–80)	116/25	Mean (range) 42.5 (1.3–246)	LNM, LVI, CIS, ACT
Manoharan et al. [5]	2010	USA	1992–2008	357	NA	185/72	NA	LNM, LVI
Canter et al. [6]	2009	USA	1988–2006	406	Mean ± SD 65.5 ± 10	NA	Mean 46.4	Age, LNM
Muramaki et al. [38]	2008	Japan	1995–2004	51	Median (range) 65 (46–74)	43/8	Median (range) 26.5 (6–102)	Age, gender, grade, LNM, LVI, CIS
Turkolmez et al. [39]	2007	Turkey	1990–2005	225	NA	NA	NA	Age, gender, LNM,,LVI
Karam et al. [40]	2007	USA	1987–2002	222	Median (IQR) 66.2(58–74/7)	177/45	Median (IQR) 36.9(13.3–79)	grade, LNM, LVI, CIS, ACT

m/f: male/femal; SD: standard deviation; NA, data not applicable; LNM: lymph node metastasis, LVI: lymphovascular invasion, STSM: soft tissue surgical margin, CIS: carcinoma in situ, ACT: adjuvant chemotherapy

**Table 2:** Tumor characteristics of all studies included in the meta-analysis

Study	Staging system	Grading system	LNM + / LNM -	CIS + /CIS-	Stage 1–2/ 3–4	Grade 1–2/ 3	STSM +/ STSM-	LVI+/ LVI-	ACT administered/ no ACT
Mayr et al. [12]	2010 TNM	NA	132/368	171/329	276/224	NA	47/453	200/300	65/435
Hodgson et al. [13]	2010 AJCC	WHO	89/146	107/128	46/189	NA	58/177	149/86	47/188
Muppa et al. [14]	2010 AJCC	WHO	797/168	NA	536/429	NA	23/942	306/659	NA
Li et al. [15]	2009 TNM	WHO	NA	NA	1,676/0	685/991	NA	188/1,488	NA
Kang et al. [16]	2009 TNM	WHO/ ISUP	191/46	78/159	168/69	51/185	3/234	67/170	185/52
Gorgel et al. [17]	2009 TNM	WHO	53/96	NA	74/75	29/119	NA	44/105	NA
Andera et al. [18]	2009 TNM	WHO	277/171	NA	160/288	12/436	NA	185/163	40/408
Crozier et al. [19]	2009 TNM	NA	NA	NA	155/65	NA	17/203	NA	NA
Morizawa et al. [20]	2009 TNM	WHO	22/88	NA	56/54	NA	13/97	31/79	NA
Liu et al. [21]	2002 TNM	WHO	63/233	NA	194/102	75/221	NA	NA	75/221
Bostrom et al. [22]	2002 TNM	WHO	301/280	NA	407/174	109/472	NA	NA	77/504
Alimi et al. [23]	NA	NA	195/136	NA	140/191	NA	40/291	NA	11/320
Soave et al. [24]	2002 TNM	WHO	138/379	187/330	0/293	30/263	261/32	NA	101/416
Raza et al. [25]	2002 TNM	WHO	33/484	NA	260/257	NA	55/462	NA	134/383
Ozcan et al. [26]	2002 TNM	WHO	42/244	19/267	162/124	96/190	18/268	51/235	NA
Kwon et al. [27]	2010 AJCC	WHO	556/190	189/557	386/338	108/636	23/723	310/436	176/570
Kanatani et al. [8]	2009 AJCC	WHO	18/43	NA	8/53	7/54	7/54	51/10	61
Ferro et al. [28]	2009 TNM	WHO	266/771	162/875	813/224	115/922	NA	NA	301/736
Booth et al. [29]	NA	NA	821/2,123	NA	807/1,995	NA	377/2,567	1,451/1,493	537/2,407
Albisinni et al. [30]	NA	NA	387/116	NA	291/212	NA	29/474	NA	NA
Kawai et al. [31]	NA	NA	65/19	NA	NA	21/60	NA	49/35	NA
Kaushik et al. [32]	2010 TNM	WHO	53/75	NA	0/128	NA	20/108	NA	NA
Brunocilla et al. [33]	2009 TNM	WHO	207/75	NA	147/135	66/216	NA	115/167	91/191
Aziz et al. [3]	2009 TNM	WHO	59/91	72/78	57/93	11/139	NA	85/65	35/115
Otto et al. [34]	2002 TNM	ISUP	640/1,843	765/1,718	1,377/1,106	829/1,654	NA	876/1,607	245/2,138
Gondo et al. [35]	NA	NA	21/173	NA	108/86	21/173	20/174	99/95	48/146
Yafi et al. [36]	1997 TNM	WHO	544/1,559	NA	1,164/1,123	NA	173/1,843	NA	401/1,662
Faba et al. [37]	2002 AJCC	WHO	7/134	33/108	141/0	132/9	NA	28/113	15/126
Manoharan et al. [5]	1997 TNM	WHO	73/284	136/221	224/133	54/293	NA	105/252	NA
Canter et al. [6]	1997 TNM	WHO	NA	NA	368/38	NA	NA	40/366	NA
Muramaki et al. [38]	2002 TNM	WHO	26/25	7/44	6/45	7/44	NA	41/10	51/0
Turkolmez et al. [39]	1997 TNM	WHO	131/94	NA	157/68	NA	NA	NA	NA
Karam et al. [40]	2002 TNM	WHO	65/160	93/132	107/119	17/209	NA	101/124	60/165

SD: standard deviation; NA: data not applicable; AJCC: American Joint Committee on Cancer classification; WHO/ ISUP: World Health Organization/International Society of Urological Pathology classification; LNM: lymph node metastasis, LVI: lymphovascular invasion, STSM: soft tissue surgical margin, CIS: carcinoma in situ, ACT: adjuvant chemotherapy

### Meta-analysis

**Fig. 2:**
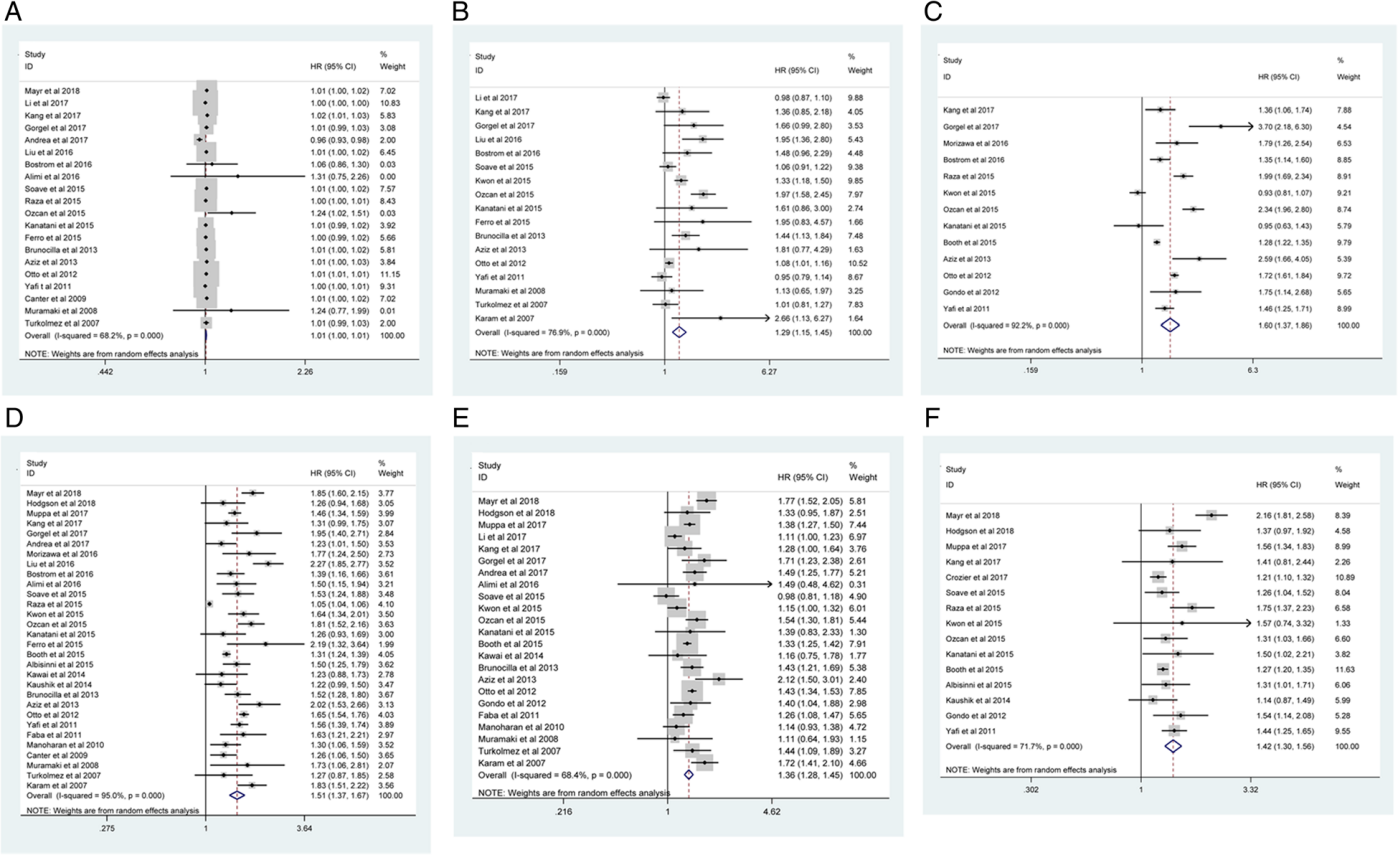
Meta-analysis of studies that examined the association between: (**2A**) advanced age, (**2B**) higher tumor grade, (**2C**) higher pathological stage, (**2D**) LNM, (**2E**) LVI, (**2F**) STSM and CSS following radical cystectomy (RC)

**Fig. 3:**
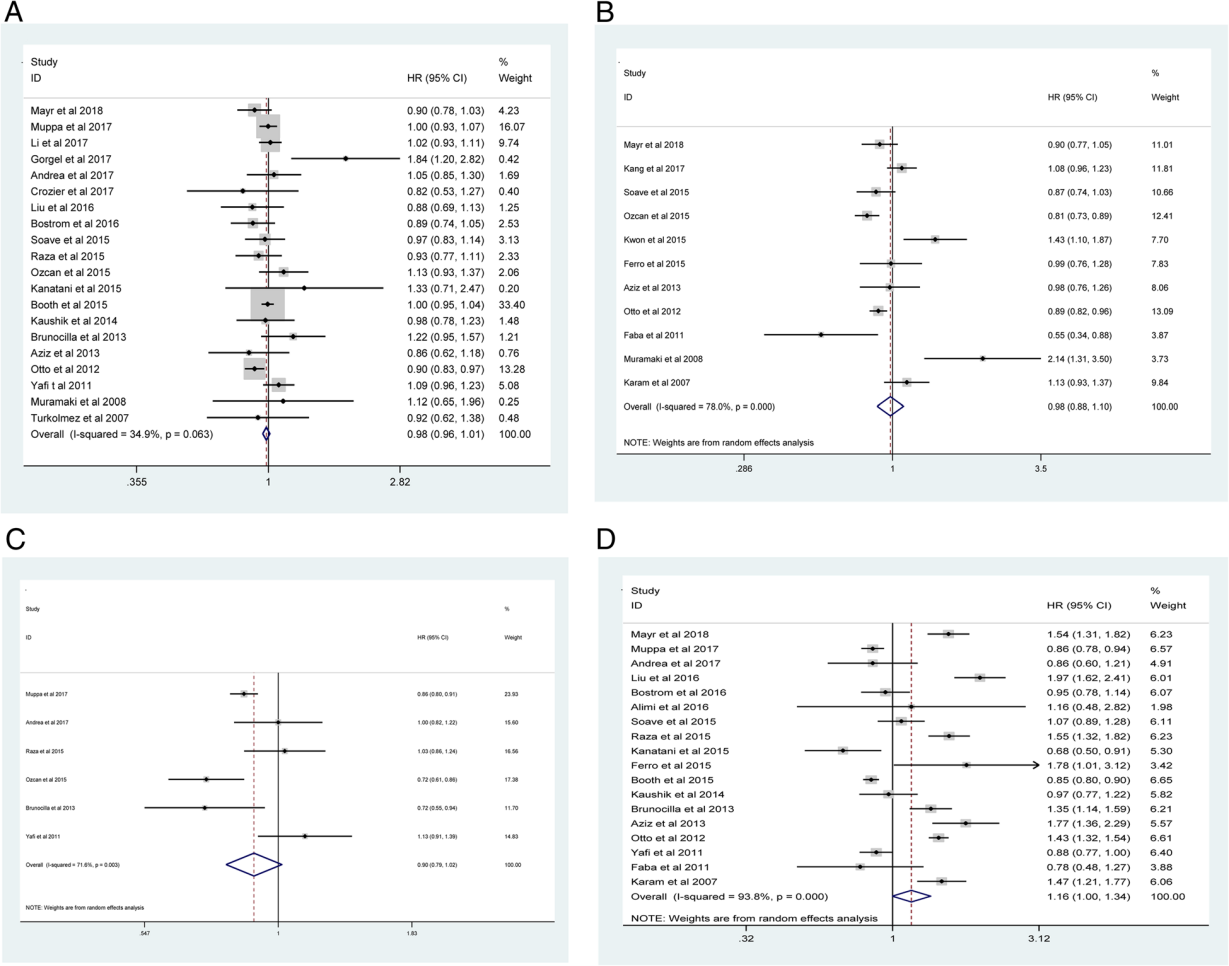
Meta-analysis of studies that examined the association between: (**3A**) gender, (**3B**) CIS, **(3C)** histology, (**3D**) ACT and CSS following radical cystectomy (RC)

Our meta-analysis demonstrated that advanced age (old vs. young: pooled HR = 1.01; 95% CI: 1.00–1.01; *P* < 0.001; *I*^*2*^ = 68.2%, *P*_*heterogeneity*_ < 0.001; Fig. 2A), higher tumor grade (3 vs. 1/2: pooled HR = 1.29; 95% CI: 1.15–1.45; *P*_*heterogeneity*_ < 0.001; *I*^*2*^ = 76.9%, *P*_*heterogeneity*_ < 0.001; Fig. 2B), higher pathological stage (3/ 4 vs. 1/ 2: pooled HR = 1.60; 95% CI: 1.37–1.86; *P* < 0.001; *I*^*2*^ = 92.2%, *P*_*heterogeneity*_ < 0.001; Fig. 2C), LNM (positive vs. negative: pooled HR = 1.51; 95% CI: 1.37–1.67; *P*_*heterogeneity*_ < 0.001; *I*^*2*^ = 95%, *P* < 0.001; Fig. 2D), LVI (positive vs. negative: pooled HR = 1.36; 95% CI: 1.28–1.45; P < 0.001; *I*^*2*^ = 68.4%, *P*_*heterogeneity*_ < 0.001; Fig. 2E), and STSM (positive vs. negative: pooled HR = 1.42; 95% CI: 1.30–1.56; P < 0.001; *I*^*2*^ = 71.7%, *P*_*heterogeneity*_ < 0.001; Fig. 2F) in BCa were associated with poor CSS. However, no significant correlations were observed regarding gender (male vs. female: pooled HR = 0.98; 95% CI: 0.96–1.01; *P* = 0.278; *I*^*2*^ = 34.9%, *P*_*heterogeneity*_ = 0.036; Fig. 3A), CIS (positive vs. negative: pooled HR = 0.98; 95% CI: 0.88–1.10; *P* = 0.753; *I*^*2*^ = 78%, *P*_*heterogeneity*_ < 0.001; Fig. 3B), histology (TCC vs variant: pooled HR = 0.90; 95% CI: 0.79–1.02; *P* = 0.089; *I*^*2*^ = 71.6%, *P*_*heterogeneity*_ = 0.003; Fig. 3C) or ACT (yes vs. no: pooled HR = 1.16; 95% CI: 1.00–1.34; *P* = 0.054; *I*^*2*^ = 93.8%, *P*_*heterogeneity*_ < 0.001; Fig. 3D).

To explore the source of heterogeneity for advanced age, tumor grade, pathological stage, LNM, LVI, STSM, CIS and ACT, their significance levels were further evaluated via subgroup analysis based on geographical region (Asia vs. non-Asia), year of publication (≥2015 vs. < 2015), number of patients (≥500 vs. < 500) and median follow-up (≥36 months vs. < 36 months). Because few studies were included in the histology group, no subgroup analysis was conducted for histology. Table 3 presents the subgroup analysis results for CSS. Notably, we observed a significant decline in heterogeneity for CSS in some categories, such as in articles published before 2015, studies with sample sizes of < 500 cases and median follow-ups of < 36 months. The subgroup analysis results were consistent with the primary findings.

**Table 3:** Summary and subgroup results of the association between common clinicopathological characteristics and BCa

Analysis specification	No. of studies	Study heterogeneity		HR(95% CI)	*P*-Value	Analysis specification	No. of studies	Study heterogeneity		HR(95% CI)	*P*-Value
		*I*^*2*^ (%) *P*_*heterogeneity*_						*I*^*2*^ (%) *P*_*heterogeneity*_			
Advanced age						LVI					
Overall	20	68.2	< 0.001	1.01(1.00,1.01)	< 0.001	Overall	23	68.4	< 0.001	1.36(1.28,1.45)	< 0.001
Geographical region						Geographical region					
Asia	8	59.3	0.016	1.01(1.00,1.02)	0.023	Asia	11	44.8	0.053	1.30(1.17,1.43)	< 0.001
non-Asia	12	68.5	< 0.001	1.01(1.00,1.01)	0.004	non-Asia	12	74	< 0.001	1.40(1.30,1.52)	< 0.001
Year of publication						Year of publication					
≥ 2015	13	72.4	< 0.001	1.01(1.00,1.01)	0.037	≥ 2015	13	74.8	< 0.001	1.34(1.22,1.46)	< 0.001
< 2015	7	39.4	0.129	1.01(1.00,1.01)	< 0.001	< 2015	10	48.9	0.040	1.40(1.28,1.54)	< 0.001
No. of patients						No. of patients					
≥ 500	8	71.9	0.001	1.01(1.00,1.01)	0.002	≥ 500	10	80.6	< 0.001	1.30(1.19,1.42)	< 0.001
< 500	12	65	0.001	1.01(1.00,1.02)	0.074	< 500	13	39.1	0.073	1.44(1.32,1.57)	< 0.001
Median follow-up						Median follow-up					
≥ 36 months	8	74.8	< 0.001	1.00(0.99,1.01)	0.736	≥ 36 months	7	72.1	0.001	1.33(1.19,1.48)	< 0.001
< 36 months	9	35.5	0.134	1.01(1.00,1.01)	< 0.001	< 36 months	10	74.3	< 0.001	1.43(1.26,1.62)	< 0.001
Grade						STSM					
Overall	17	76.9	< 0.001	1.29(1.15,1.45)	< 0.001	Overall	15	71.7	< 0.001	1.42(1.30,1.56)	< 0.001
Geographical region						Geographical region					
Asia	9	82.6	< 0.001	1.37(1.12,1.68)	0.002	Asia	7	0	0.650	1.26(1.17,1.36)	< 0.001
non-Asia	8	57.9	0.002	1.17(1.03,1.34)	0.020	non-Asia	8	55.5	< 0.001	1.46(1.27,1.67)	< 0.001
Year of publication						Year of publication					
≥ 2015	10	81.6	< 0.001	1.41(1.17,1.70)	< 0.001	≥ 2015	12	76.1	< 0.001	1.44(1.29,1.61)	< 0.001
< 2015	7	54.4	0.041	1.13(0.98,1.31)	0.085	< 2015	3	29.3	0.243	1.38(1.19,1.60)	< 0.001
No. of patients						No. of patients					
≥ 500	7	71.1	0.002	1.11(0.99,1.23)	0.072	≥ 500	10	78.1	< 0.001	1.50(1.32,1.69)	< 0.001
< 500	10	60.5	0.007	1.53(1.25,1.87)	< 0.001	< 500	5	0	0.745	1.22(1.13,1.32)	< 0.001
Median follow-up						Median follow-up					
≥ 36 months	6	88.3	< 0.001	1.45(1.15,1.84)	0.002	≥ 36 months	6	34.3	0.179	1.43(1.26,1.62)	< 0.001
< 36 months	8	36	0.141	1.10(0.98,1.23)	0.113	< 36 months	6	75	0.001	1.53(1.27,1.84)	< 0.001
Stage						CIS					
Overall	13	92.2	< 0.001	1.60(1.37,1.86)	< 0.001	Overall	11	78	< 0.001	0.98(0.88,1.10)	0.753
Geographical region						Geographical region					
Asia	7	93.1	< 0.001	1.61(1.10,2.63)	0.013	Asia	4	91	< 0.001	1.19(0.88,1.61)	0.251
non-Asia	5	92.5	< 0.001	1.60(1.35,1.90)	< 0.001	non-Asia	7	43.3	0.102	0.92(0.84,1.01)	0.068
Year of publication						Year of publication					
≥ 2015	9	92.7	< 0.001	1.54(1.25,1.90)	< 0.001	≥ 2015	6	79.2	< 0.001	0.97(0.84,1.12)	0.709
< 2015	4	58	0.068	1.70(1.45,1.98)	< 0.001	< 2015	5	81.2	< 0.001	1.01(0.80,1.28)	0.939
No. of patients						No. of patients					
≥ 500	8	93.1	< 0.001	1.47(1.24,1.73)	< 0.001	≥ 500	5	67.3	0.016	0.96(0.84,1.09)	0.520
< 500	5	87.2	< 0.001	1.92(1.29,2.87)	0.001	< 500	6	84.6	< 0.001	1.00(0.81,1.24)	0.971
Median follow-up						Median follow-up					
≥ 36 months	4	96.4	< 0.001	1.55(1.02,2.37)	0.042	≥ 36 months	2	93.5	< 0.001	1.06(0.60,1.86)	0.838
< 36 months	6	65.9	0.012	1.62(1.37,1.92)	< 0.001	< 36 months	8	68.4	0.002	0.96(0.84,1.08)	0.487
LNM						ACT					
Overall	30	95	< 0.001	1.51(1.37,1.67)	< 0.001	Overall	18	93.8	< 0.001	1.16(1.00,1.34)	0.054
Geographical region						Geographical region					
Asia	11	61.2	0.004	1.58(1.38,1.81)	< 0.001	Asia	2	97.1	< 0.001	1.16(0.41,3.31)	0.775
non-Asia	19	96.2	< 0.001	1.48(1.32,1.66)	< 0.001	non-Asia	16	93.4	< 0.001	1.15(0.99,1.34)	0.063
Year of publication						Year of publication					
≥ 2015	18	94.9	< 0.001	1.52(1.34,1.71)	< 0.001	≥ 2015	11	93.4	< 0.001	1.12(0.92,1.37)	0.243
< 2015	12	58.6	0.005	1.50(1.38,1.64)	< 0.001	< 2015	7	89.6	< 0.001	1.21(0.99,1.48)	0.053
No. of patients						No. of patients					
≥ 500	14	98.9	< 0.001	1.48(1.29,1.70)	< 0.001	≥ 500	9	95.7	< 0.001	1.13(0.94,1.37)	0.201
< 500	16	69.1	< 0.001	1.53(1.38,1.71)	< 0.001	< 500	9	86.3	< 0.001	1.18(0.93,1.50)	0.177
Median follow-up						Median follow-up					
≥ 36 months	11	95.3	< 0.001	1.47(1.24,1.74)	< 0.001	≥ 36 months	8	92.4	< 0.001	1.16(0.91,1.49)	0.228
< 36 months	13	49.4	0.022	1.61(1.49,1.74)	< 0.001	< 36 months	9	89.9	< 0.001	1.20(0.99,1.46)	0.065

### Sensitivity analysis

The pooled HR for CSS for advanced age ranged from 1.01 (95% CI:1.00–1.01) to 1.01 (95% CI:1.00–1.01), for gender ranged from 0.98 (95% CI: 0.94–1.02) to 0.99 (95% CI: 0.99–1.04), for tumor grade ranged from 1.25 (95% CI: 1.11–1.41) to 1.34 (95% CI: 1.16–1.54), for pathological stage ranged from 1.53 (95% CI: 1.31–1.79) to 1.68 (95% CI: 1.45–1.95), for LNM ranged from 1.49 (95% CI: 1.35–1.64) to 1.52 (95% CI: 1.37–1.68), for LVI ranged from 1.34 (95% CI: 1.26–1.42) to 1.38 (95% CI: 1.30–1.47), for STSM ranged from 1.34 (95% CI: 1.26–1.43) to 1.44 (95% CI: 1.29–1.61), for CIS ranged from 0.95 (95% CI: 0.86–1.05) to 1.01 (95% CI: 0.89–1.14), for histology ranged from 0.86 (95% CI: 0.76–0.97) to 0.94 (95% CI: 0.82–1.07), and for ACT ranged from 1.12 (95% CI: 0.97–1.29) to 1.19 (95% CI: 1.02–1.38) (Additional file 1: Figure S1).These results indicated that our findings were reliable and robust.

### Publication bias

**Fig. 4:**
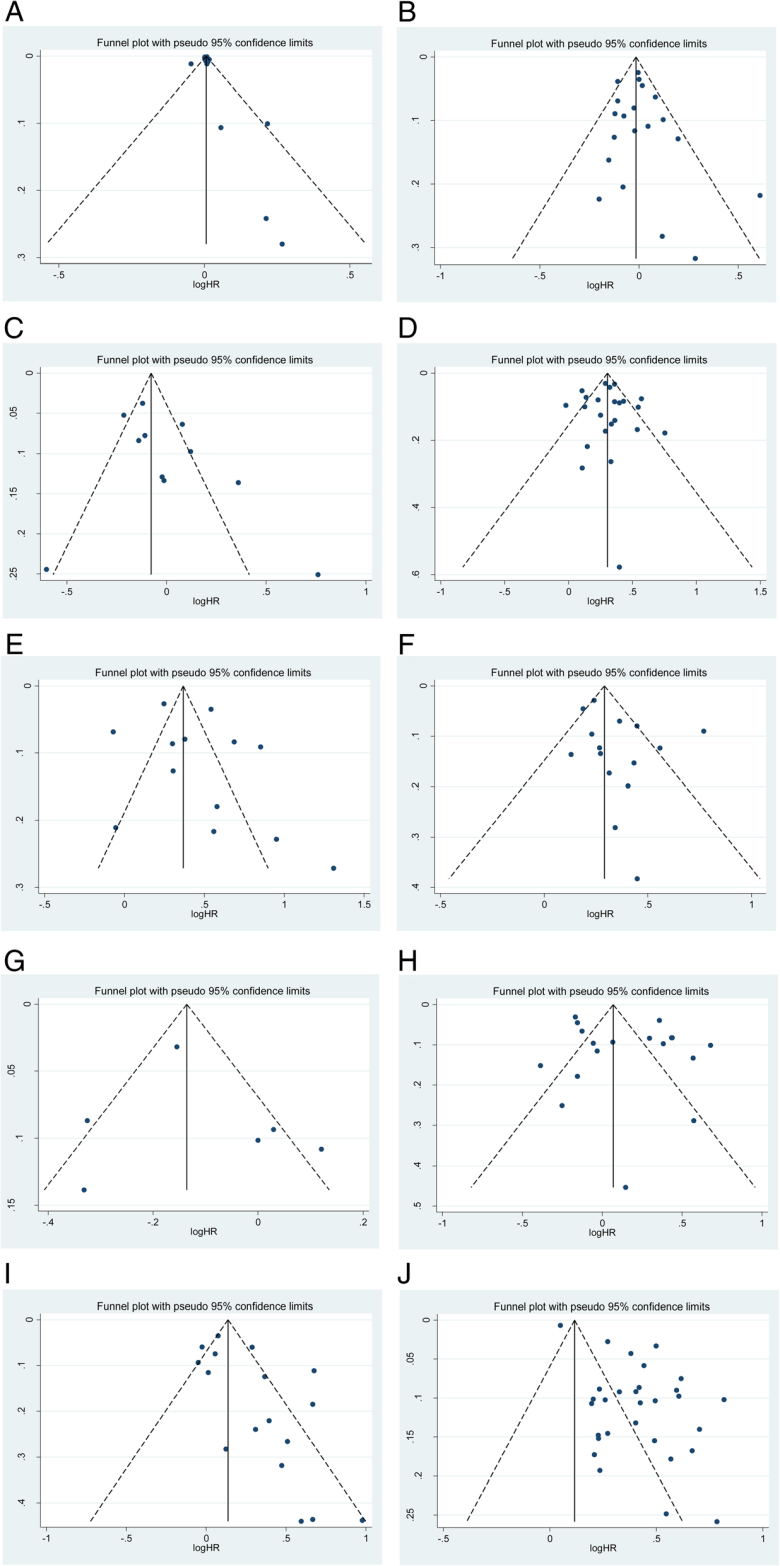
Funnel plots for the publication bias test. Each point represents a separate study for the indicated association. The vertical line represents the mean effects size: (**4A**) advanced age; (**4B**) gender; (**4C**) CIS; (**4D**) LVI; (**4E**) pathological stage; (**4F**) STSM; (**4G**) histology; **(4H)** ATC; (**4I**) tumor grade and (**4 J**) LNM

Figure 4 shows the funnel plots for publication bias. Egger’s test demonstrated that no publication bias existed regarding advanced age (p Egger = 0.427, Fig. 4A), gender (p Egger = 0.487, Fig. 4B), CIS (p Egger = 0.172, Fig. 4C), LVI (p Egger = 0.797, Fig. 4D), pathological stage (p Egger = 0.330, Fig. 4E), STSM (p Egger = 0.134, Fig. 4F), histology (p Egger = 0.648, Fig. 4G) and ATC (p Egger = 0.266, Fig. 4H). However, publication biases were found for tumor grade (p Egger = 0.023, Fig. 4I) and LNM (p Egger< 0.001, Fig. 4J), suggesting that publication bias may have played a potential role in tumor grade and LNM.

## Discussion

Despite modern advancements in surgical techniques, the oncological outcomes of BCa remains poor. The 5-yr overall survival rates were only 60% according to a multicenter database [41]. Determining the probability of CSS after RC is difficult because it can vary according to the different clinical features and various tumor characteristics. The traditional clinicopathological features, such as sex [34], pathological tumor stage or grade [25] and LNM [6], have been identified as important parameters with prognostic predictive value and contribute to postoperative clinical decision making based on some nomograms.

Currently, the TNM staging system, which is based on pathological tumor stage and grade, tumor histological subtype, and lymph node status [42] is the most commonly used preoperative model for predicting CSS in BCa patients. Another predictive model is the European Organisation for the Research and Treatment of Cancer (EORTC) risk stratification scheme [43], which uses grade (World Health Organization [WHO] 1973), stage, CIS, multiplicity, size and previous recurrence rate to determine the risk of CSS after RC. Although these two traditional prognostic models have been externally validated, significant variations were founded in some studies. Variations in tumor outcomes may have been related to the heterogeneity of BCa biology and different clinicopathological features in patients with BCa.

Tumor markers that can accurately predict the oncological outcomes in BCa patients when applied with other pathological parameters are essential for clinical decision making. Some published studies on molecular biomarkers, such as luminal and basal subtypes [44], the gene alterations nuclear matrix protein number 22 [45], and the bladder tumor antigen (BTA) stat test [46], have been adopted in recent years to improve diagnosing and managing patients receiving RC. However, none of these biomarkers have been shown to be sufficiently sensitive or specific in predicting survival outcomes. Therefore, in this study, we exploited more validated prognostic factors, including clinical variables (age, gender), pathological information (tumor stage and grade, LNM and STSM, LVI, CIS, and histology), and whether adjuvant therapy (ACT) was received for predicting CSS in BCa patients.

This is the first study to systematically assess the association between ten clinicopathological features and CSS of BCa in a single study. To improve the statistical power and provide more credible results, 33 cohort studies with a large combined sample size of 19,702 BCa patients who underwent RC were pooled in our study. Strictly adhering to the inclusion and exclusion criteria, we extracted the raw data from the relevant studies. The results revealed that advanced age, higher tumor grade, LNM, LVI, and positive STSM significantly predicted the CSS of BCa patients (all *P* ≤ 0.05). Hence, these clinicopathological findings were independent risk factors in this meta-analysis. Besieds, all the results were reliable and robust via the subgroup and sensitivity analyses.

Interestingly, our results indicated that gender, CIS, histology and ACT may not be associated with CSS. Studies on gender, histology and CIS as prognostic factors for BCa patients have stimulated considerable interest, but the results remain controversial and ambiguous for managing BCa. Some investigators reported that gender and CIS had independent prognostic significance [14, 34, 47], while others considered that gender and CIS may not be significant factors in determining terminal prognosis compared with other widely used prognostic indicators [18, 48, 49]. Additionally, administering ACT after RC in patients with high-risk BCa remains a challenge for clinical urologists. Despite numerous studies being published, no level 1 evidence has demonstrated that ATC confers a significant survival benefit to BCa patients after RC [50]. In the present study, rigorous data analysis indicated that these three factors may not affect the CSS prognosis of patients with BCa.

Although this was a comprehensive meta-analysis, the present study had several limitations. First, most included studies were retrospective cohort studies, and data extracted from those studies may have led to inherent bias. Thus, a prospective multicenter trial providing more definite answers is needed. Second, substantial heterogeneity was observed in some studies. Although we found no possible source of heterogeneity after several subgroup analyses, the conclusions drawn from this meta-analysis should be approached with caution. However, the pooled results in most of the subgroup analyses were consistent with the overall findings. Third, the studies retrieved for our analysis were limited to those published in English, which may result in a language bias. Studies with negative results are not often published in English-language journals [51]; thus, our research may contain some publication bias.

## Conclusions

In summary, the data from this meta-analysis indicate that BCa patients with advanced age, higher tumor grade, LNM, LVI, and positive STSM are likely to have poorer CSS, suggesting that these parameters may be independent indicators of BCa in patients following RC. In contrast with what is seen clinical practice, gender, CIS, histology and postoperative ACT were not predictors of CSS in patients with BCa. We identified significant patient-specific (age) and tumor-specific (higher tumor grade, LNM, LVI, and positive STSM) predictors of CSS to propose a risk-based strategy for choosing surveillance and postoperative treatment options. Despite our rigorous systematic approach, further large, prospective studies are needed to confirm our findings considering the inherent limitations of the included studies.

## Additional files

Additional file 1: **Figure S1** Sensitivity analysis for: (**S1A**) advanced age; (**S1B**) gender; (**S1C**) tumor grade; (**S1D**) pathological stage; (**S1E**) LNM; (**S1F**) LVI; (**S1G**) STSM; (**S1H**) CIS; (**S1I**) histology; (**S1J**) ACT. (TIF 10703 kb)

Additional file 2: **Table S1** Quality assessment of the cohort studies included in this meta-analysis. (DOCX 57 kb)

## Data Availability

All data generated or analyzed during the present study are included in this published article (and its supplementary information files).

## Abbreviations

ACT: Adjuvant chemotherapy
BCa: Bladder cancer
CIS: Concomitant carcinoma in situ
CIs: Confidence intervals
CSS: Cancer-specific survival
EORTC: European Organisation for the Research and Treatment of Cancer
HRs: Hazard ratios
LNM: Lymph node metastasis
LVI: Lymphovascular invasion
NOS: Newcastle-Ottawa scale
PRISMA: Preferred Reporting Items for Systematic Reviews and Meta-Analyses
RC: Radical cystectomy
STSM: Soft tissue surgical margin
TCC: Transitional cell cancer
