# Supplementary figures and images for: Clinicopathological factors in bladder cancer for cancer-specific survival outcomes following radical cystectomy: a systematic review and meta-analysis
Source: BMC Cancer. 2019 Jul 19;19:716. doi: 10.1186/s12885-019-5924-6 (PMC6642549; doi:10.1186/s12885-019-5924-6)

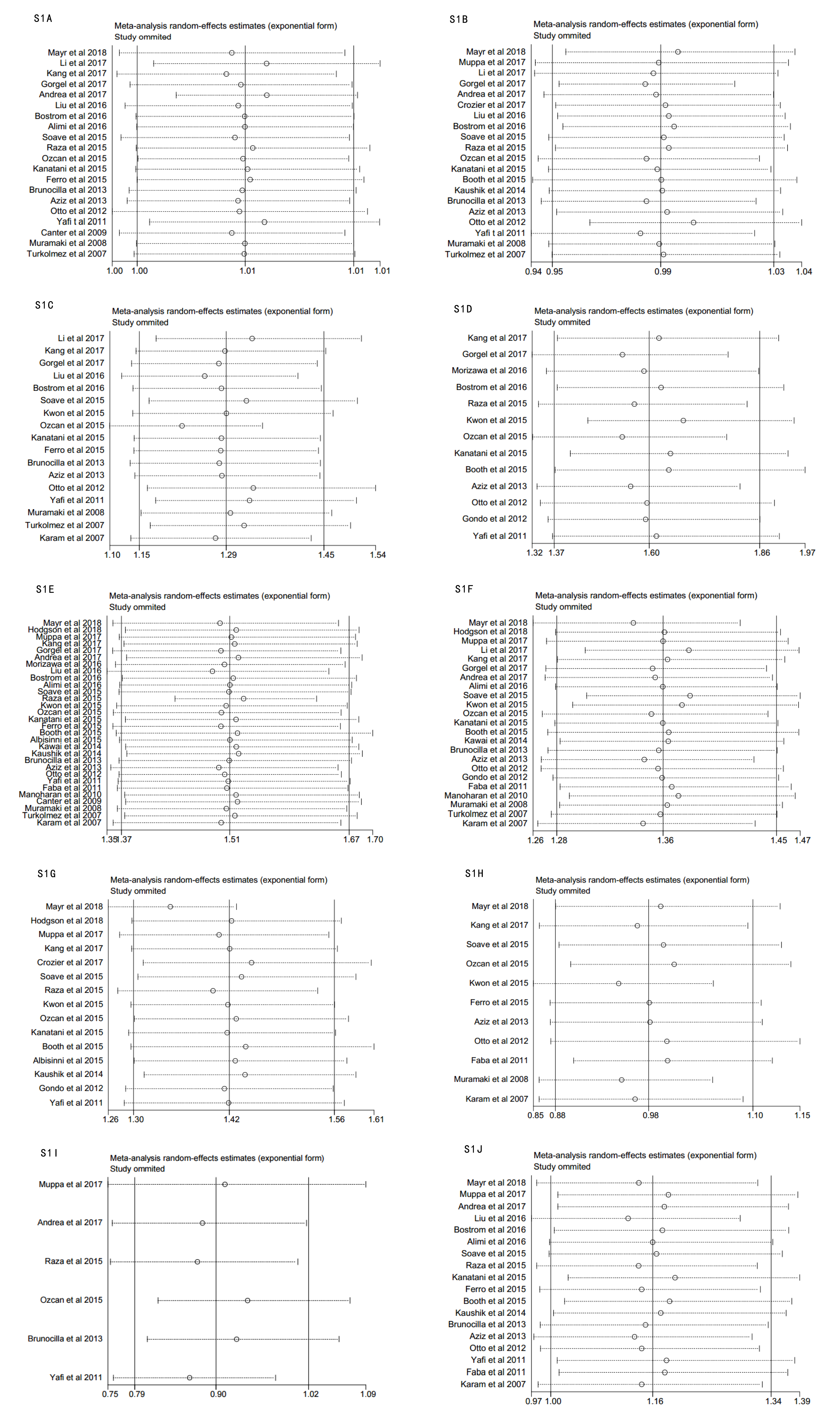

Supplement: Supplementary file 1 — Figure S1 Sensitivity analysis for: (S1A) advanced age; (S1B) gender; (S1C) tumor grade; (S1D) pathological stage; (S1E) LNM; (S1F) LVI; (S1G) STSM; (S1H) CIS; (S1I) histology; (S1J) ACT. (TIF 10703 kb) [file 12885_2019_5924_MOESM1_ESM.tif]
