# Supplementary material for: Clinicopathological factors in bladder cancer for cancer-specific survival outcomes following radical cystectomy: a systematic review and meta-analysis
Source: BMC Cancer. 2019 Jul 19;19:716. doi: 10.1186/s12885-019-5924-6 (PMC6642549; doi:10.1186/s12885-019-5924-6)
Supplement: Supplementary file 2 — Table S1 Quality assessment of the cohort studies included in this meta-analysis. (DOCX 57 kb) [file 12885_2019_5924_MOESM2_ESM.docx]

**Table S1. Quality assessment of cohort studies included in this meta- analysis**

| **Study** | **Representativeness of the exposed cohort** | **Selection of the unexposed cohort** | **Ascertainment of exposure** | **Outcome of interest not present at start of study** | **Control for important factor or additional factor** | **Outcome assessment** | **Follow-up long enough for outcomes to occur** | **Adequacy of follow-up of cohort** | **Total quality scores** |
| --- | --- | --- | --- | --- | --- | --- | --- | --- | --- |
| Mayr et al[[12](#_ENREF_12)] | ★ | ★ | ★ | ★ | ★ | ★ | ★ | ★ | 8 |
| Hodgson et al[[13](#_ENREF_13)] | ★ | ★ | ★ | ★ | ★ | ★ | ★ | ★ | 8 |
| Muppa et al[[14](#_ENREF_14)] | ★ | ★ | ★ | ★ | ★★ | ★ | ★ | ★ | 9 |
| Li et al[[15](#_ENREF_15)] | ★ | ★ | — | ★ | ★★ | ★ | ★ | ★ | 8 |
| Kang et al[[16](#_ENREF_16)] | ★ | ★ | ★ | ★ | ★★ | ★ | ★ | — | 8 |
| Gorgel et al[[17](#_ENREF_17)] | ★ | ★ | ★ | ★ | ★ | ★ | ★ | — | 7 |
| Andera et al[[18](#_ENREF_18)] | ★ | ★ | ★ | ★ | ★★ | ★ | ★ | ★ | 0 |
| Crozier et al[[19](#_ENREF_19)] | ★ | ★ | ★ | ★ | ★ | ★ | ★ | — | 7 |
| Morizawa et al[[20](#_ENREF_20)] | ★ | ★ | — | ★ | ★ | ★ | ★ | ★ | 7 |
| Liu et al[[21](#_ENREF_21)] | ★ | ★ | — | ★ | ★ | ★ | ★ | ★ | 7 |
| Bostrom et al[[22](#_ENREF_22)] | ★ | ★ | ★ | ★ | ★ | ★ | ★ | ★ | 8 |
| Alimi et al[[23](#_ENREF_23)] | ★ | ★ | ★ | ★ | ★★ | ★ | ★ | ★ | 9 |
| Soave et al[[24](#_ENREF_24)] | ★ | ★ | ★ | ★ | ★ | ★ | ★ | ★ | 8 |
| Raza et al[[25](#_ENREF_25)] | ★ | ★ | ★ | ★ | ★ | ★ | ★ | ★ | 8 |
| Ozcan et al[[26](#_ENREF_26)] | ★ | ★ | — | ★ | ★★ | ★ | ★ | ★ | 8 |
| Kwon et al[[27](#_ENREF_27)] | ★ | ★ | — | ★ | ★ | ★ | ★ | ★ | 7 |
| Kanatani et al[[8](#_ENREF_8)] | ★ | ★ | ★ | ★ | ★ | ★ | ★ | ★ | 8 |
| Ferro et al[[28](#_ENREF_28)] | ★ | ★ | ★ | ★ | ★ | ★ | ★ | ★ | 8 |
| Booth et al[[29](#_ENREF_29)] | ★ | ★ | ★ | ★ | ★ | ★ | ★ | — | 7 |
| Albisinni et al[[30](#_ENREF_30)] | ★ | ★ | — | ★ | ★ | ★ | ★ | ★ | 7 |
| Kawai et al[[31](#_ENREF_31)] | ★ | ★ | ★ | ★ | ★ | ★ | ★ | ★ | 8 |
| Kaushik et al[[32](#_ENREF_32)] | ★ | ★ | ★ | ★ | ★ | ★ | — | ★ | 7 |
| Brunocilla et al[[33](#_ENREF_33)] | ★ | ★ | ★ | ★ | ★★ | ★ | ★ | ★ | 9 |
| Aziz et al[[3](#_ENREF_3)] | ★ | ★ | ★ | ★ | ★★ | ★ | ★ | ★ | 9 |
| Otto et al[[34](#_ENREF_34)] | ★ | ★ | — | ★ | ★★ | ★ | ★ | ★ | 8 |
| Gondo et al[[35](#_ENREF_35)] | ★ | ★ | ★ | ★ | ★ | ★ | ★ | ★ | 8 |
| Yafi et al[[36](#_ENREF_36)] | ★ | ★ | — | ★ | ★ | ★ | ★ | ★ | 7 |
| Faba et al[[37](#_ENREF_37)] | ★ | ★ | ★ | ★ | ★ | ★ | ★ | ★ | 8 |
| Manoharan et al[[5](#_ENREF_5)] | ★ | ★ | ★ | ★ | ★ | ★ | ★ | — | 7 |
| Canter et al[[6](#_ENREF_6)] | ★ | ★ | — | ★ | ★★ | ★ | ★ | ★ | 8 |
| Muramaki et al[[38](#_ENREF_38)] | ★ | ★ | ★ | ★ | ★ | ★ | ★ | ★ | 8 |
| Turkolmez et al[[39](#_ENREF_39)] | ★ | ★ | ★ | ★ | ★ | ★ | ★ | — | 7 |
| Karam et al[[40](#_ENREF_40)] | ★ | ★ | — | ★ | ★★ | ★ | ★ | ★ | 8 |
